# Supplementary material for: The piston Riemann problem in a photon superfluid
Source: Nat Commun. 2022 Jun 6;13:3137. doi: 10.1038/s41467-022-30734-5 (PMC9170689; doi:10.1038/s41467-022-30734-5)
Supplement: Supplementary file 1 — Supplementary Information [file 41467_2022_30734_MOESM1_ESM.pdf]

The piston Riemann problem in a photon superfluid: Supplementary Information

Abdelkrim Bendahmane[1], Gang Xu [1], Matteo Conforti[1], Alexandre Kudlinski[1], Arnaud Mussot[1], and Stefano Trillo[2]
[1] *Univ. Lille, CNRS, UMR 8523 - PhLAM - Physique des Lasers Atomes et Molécules, F-59000 Lille, France and*
[2] *Department of Engineering, University of Ferrara, Via Saragat 1, 44122 Ferrara, Italy*

Here we present supplementary information for the paper "The piston Riemann problem in a photon superfluid", discussing specific details that support the theory (Supplementary notes 1 and 2), additional numerical results (Supplementary note 3), and deepening some technical aspects of the experimental setup (Supplementary note 4).

SUPPLEMENTARY NOTE 1: PISTON ACTION AND CLASSICAL SHOCK WAVES

We recall here the constraints for the appearance of piston-driven classical shock waves (CSWs), as derived from the dispersionless limit [Eqs. (2-3) of main text, dropping the RHS] on the basis of general theory of shock waves [1, 2]. To this purpose the resulting system of equations can be conveniently cast in the general form of a 2×2 conservation law

$$\mathbf{q}_z + \mathbf{f}_t(\mathbf{q}) = 0, \tag{S1}$$

which physically express conservation of mass and momentum, when we set $\mathbf{q} = [\rho, \rho u]^T$, and consider the flux to be defined as $\mathbf{f} = [f_1(\rho, u), f_2(\rho, u)]^T = [\rho u, \rho u^2 + \rho^2/2]^T$ (see also [3]). Across a discontinuous solution such as a CSW, the conservation law (S1) locally reduces to the well known Rankine-Hugoniot (RH) condition $V_s = \frac{\mathbf{f}(\mathbf{q_L})-\mathbf{f}(\mathbf{q_R})}{\mathbf{q_L}-\mathbf{q_R}}$ [1–4], which links the shock velocity $V_s = dt/dz$ to the quantities evaluated on the left and right of the jump, denoted by the subscripts L, R, respectively (incidentally, note that, in the spirit of shock wave theory, we denote V_s as a velocity despite, dimensionally, it would be an inverse velocity; this is due to the interchanged role of time and space in Eq. (S1) compared with the standard gas-dynamic case). For the specific case of the dispersionless NLSE, the RH condition reads explicitly as (see e.g. [3])

$$V_s = \frac{u_L\rho_L - u_R\rho_R}{\rho_L - \rho_R}; V_s = \frac{u_L^2\rho_L - u_R^2\rho_R + (\rho_L^2 - \rho_R^2)/2}{u_L\rho_L - u_R\rho_R}. \tag{S2}$$

The problem of a piston set impulsively in motion at velocity V_p towards the right (Fig. 1(a) in the paper), which is compressing a gas of constant density $\overline{\rho}$ and velocity $\overline{u}$ is described by the solution of Eqs. (S1) subject to the (moving) boundary conditions $u_L = V_p$ and $(\rho_R, u_R) = (\overline{\rho}, \overline{u})$. These are compatible with a CSW, whose density on the left of the jump, say ρ_L, must be found as the only admissible root (positive and larger than ρ_R) of the following cubic equation

$$\rho_L^3 - \rho_R\rho_L^2 - \rho_L\left[\rho_R^2 + 2\rho_R(V_p - u_R)^2\right] + \rho_R^3 = 0, \tag{S3}$$

obtained by eliminating V_s in the two equations (S2). Then, the shock velocity $V_s \geq V_p$ can be calculated from any of the two Eqs. (S2). The CSW calculated in this way is supersonic with Mach number $M = V_s/\sqrt{\rho_R} \geq 1$, where $\sqrt{\rho_R}$ is the sound velocity of small disturbances. Both the Mach number M (or velocity V_s) and the shock amplitude $\Delta\rho = \rho_L - \rho_R$ grow with increasing piston velocity, as shown in Supplementary Fig. 1, where we set $\rho_R = 1$, $u_R = 0$. The sonic boundary ($M = 1$) is obtained only in the limit of non-moving piston $V_p \to 0$, which, however, consistently yields a vanishing shock amplitude ($\Delta\rho \to 0$).

When the piston is replaced by a step-like variation of input velocity, from u_L to $u_R < u_L$, over constant density $\rho_L = \rho_R = \overline{\rho}$, the admissible solution (i.e., compatible with entropy condition [1]) is composed by two CSWs that move in opposite directions and sit on a central constant intermediate state (ρ_i, u_i), which represents a constant wake of the shocks. By writing two sets of RH vectorial conditions (S2) for the left and right CSWs and imposing compatibility between them, one obtains $u_i = (u_L + u_R)/2$, as well as a cubic algebraic equation for the unknown ρ_i, which has exactly the same form of Eq. (S3), with the formal substitution $\rho_L \to \rho_i$ and $(V_p - u_R)^2 \to (\frac{u_L - u_R}{2})^2$. Then both

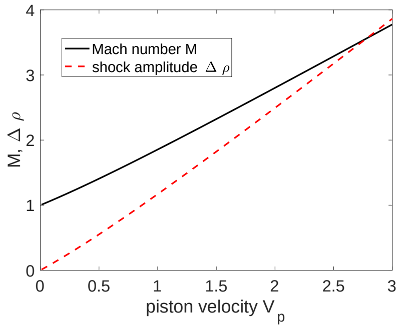

Supplementary Figure 1. Mach number M and shock amplitude $\Delta\rho$ as a function of piston velocity V_p. Without loss of generality, we fix $\rho_R = 1$, $u_R = 0$.

CSWs can be fully characterized through the RH conditions and the admissible root $\rho_i > \rho_R$ of this equation. The formal equivalence of Eq. (S3) to describe the two cases (the real piston and the Riemann problem) supports the fact that the step initial condition in the velocity correspond to two pistons pushing in opposite directions. In the specific symmetric case $u_L = -u_R = u_0$ discussed in the paper (see Fig. 1(b)), the two CSWs created by the virtual pistons are symmetric. In this case $u_i = 0$, and the cubic equation simplifies since $[(u_L - u_R)/2]^2 = u_0^2$.

The same Riemann problem for the dispersionless model can be solved by a different approach, which is far more important in view of understanding the fully dispersive case. To this end, the dispersionless model is cast in diagonal form in terms of Riemann invariant variables $r^{\pm}(z,t) = u(z,t) \pm 2\sqrt{\rho(z,t)}$, and its solution is sought for in the form of a pair of left-going and right-going so-called simple waves, such that only one between r^+ and r^- actually varies (see [5–9] for more details). The compatibility between the two simple waves allows to give explicitly ρ_i and u_i as in Eq. (4) of the main text, and to arrive at simple closed formulas for the velocities [Eqs. (5) in the main text]. The latter follow from the application of the scalar version of the RH condition to the only variable between $r^{\pm}$, which is actually varying. On one hand, such simple-wave solution gives the same result of the previous approach that leads to Eq. (S3), whenever u_0 is not too large ($u_0 \lesssim 1$). On the other hand, the constant state ρ_i, u_i calculated in this way retains its validity for the fully dispersive case, for any value of u_0. These two features become evident from the examples reported in Supplementary Fig. 2, where we compare the full NLSE dynamics (black curves) with the two different dispersionless approaches based on the vector Rankine-Hugoniot relation (blue curves) or the simple-wave solution of the conservation laws (red curves). As shown, the plateau which spontaneously forms in the full NLSE dynamics coincides with that of the simple-wave approach for both small and large values u_0 of the initial velocity jump. Ultimately, this reflects the fact the wave-pair generated in the full NLSE (the DSW pair in this case) turns out to be the dispersive regularization of the solution obtained by means of the simple-wave approach applied to the dispersionless limit. This is true in general, for both shock and rarefaction waves. When the admissible simple-wave solution of the dispersionless limit is a RW, a RW emerges in the fully dispersive NLSE, whereas a DSW emerges when the admissible solution of the dispersionless limit is a CSW (as, for instance, in the regime discussed above).

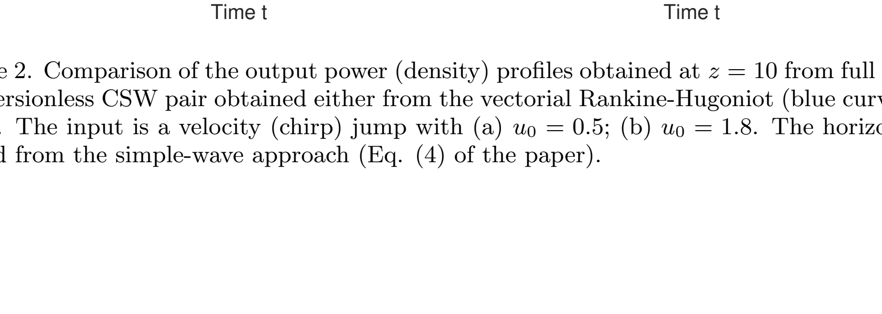

Supplementary Figure 2. Comparison of the output power (density) profiles obtained at $z = 10$ from full NLSE dynamics (black curves) with the dispersionless CSW pair obtained either from the vectorial Rankine-Hugoniot (blue curves) or the simple-wave approach (red curve). The input is a velocity (chirp) jump with (a) $u_0 = 0.5$; (b) $u_0 = 1.8$. The horizontal green dashed line stands for ρ_i obtained from the simple-wave approach (Eq. (4) of the paper).

In particular, we discuss in the next section how modulation theory allows to describe the latter regime, and specifically the details of the transition of the DSW pair from a constant wake to the fully periodic wake.

SUPPLEMENTARY NOTE 2: MODULATION THEORY

Contrary to the dispersionless limit, the solution of the full NLSE subject to the same jump initial condition is never discontinuous. Under these conditions, an accurate asymptotic description of the generated DSWs, which is valid for sufficiently large distances, can be obtained in closed form by exploiting the modulation theory based on Whitham averaging, for which we refer the reader to the detailed review in Ref. [5]. Here, we give only the essential elements and the most general formulas that we obtain to describe the primary transition DSW-c-DSW to DSW-per-DSW, peculiar to the dispersive nature of the fluid. To this end, we recall that the Riemann problem involves step initial data with generic boundaries ρ_L, u_L on the left state ($t < 0$) and ρ_R, u_R on the right state ($t > 0$). The classification of the outcome in terms of wave pairs given in Fig. 3(c) of the main paper (see also Supplementary Fig. 5) can be more conveniently made in terms of boundary initial values assumed by Riemann invariant variables (i.e., those which diagonalize the dispersionless NLSE): $r_L^\pm \equiv u_L \pm 2\sqrt{\rho_L}$ on the left ($t < 0$) and $r_R^\pm \equiv u_R \pm 2\sqrt{\rho_R}$ on the right ($t > 0$). With such general notation, the formulas reported below are valid without any restriction, though the figures refer, for sake of simplicity, to the pure piston problem ($\rho_R = \rho_0 = 1$ and $u_R = -u_L = u_0$).

The state DSW-c-DSW, of our interest here, is obtained whenever the initial step is such that $r_L^+ \geq r_R^+ \geq r_L^- \geq r_R^-$. Under such constraint, the simple wave solution of the gas dynamic equations (the dispersionless NLSE), which has self-similar structure ($r^\pm = r^\pm(\tau)$, $\tau = t/z$ being the self-similar variable), has indeed the structure illustrated in Supplementary Fig. 3(a), reported for convenience vs. time t at fixed $z = 12$. In the fully dispersive case, each DSW turns out to be a one-phase solution given by the dispersive regularization of the region where either r^+ or r^- is not constant, being multivalued. The DSW can be constructed as a slow modulation of a periodic nonlinear solution, so-called dn-oidal (or cn-oidal) wave. A set of four hyperbolic equations known as Whitham equations rules the slow evolution of a suitable combination of the four parameters of such periodic wave. In particular, the diagonalized form of Whitham equations [10] allows to construct a smooth simple wave solution of the system, which is characterized by three unknowns remaining constant (linked to the constant initial values $r_{L,R}^\pm$) and only the fourth unknown varying in self-similar way (again, as a function of $\tau = t/z$ only). This dependence in turn describes the modulation of the dn-oidal wave which constitutes the DSW. The two DSWs are then connected to the central zero-phase solution where the both $r^\pm$ are constant. The overall result of this analytical construction is shown in Supplementary Fig. 3(b). See also [5, 9] for more details on the construction of the full analytical solution.

This solution allows us to extract analytical expressions of the velocities τ that correspond to the local soliton edges (orange dashed lines in Supplementary Fig. 3(b)) and the linear edges (magenta dashed line in Supplementary Fig. 3(b)) of the two DSWs, respectively, which read for the DSW-c-DSW (see also [5, 8])

$$\tau_1 = \frac{2r_L^+ + r_R^- + r_R^+}{4} + \frac{(r_R^- - r_L^+)(r_R^+ - r_L^+)}{2r_L^+ - r_R^- - r_R^+}, \tag{S4}$$

$$\tau_2 = \frac{r_L^+ + r_R^- + 2r_R^+}{4}; \;\; \tau_3 = \frac{r_L^+ + r_R^- + 2r_L^-}{4}, \tag{S5}$$

$$\tau_4 = \frac{2r_R^- + r_L^- + r_L^+}{4} + \frac{(r_L^+ - r_R^-)(r_L^- - r_R^-)}{2r_R^- - r_L^- - r_L^+}. \tag{S6}$$

Note that τ_j, $j = 1, 2, 3, 4$ are uniquely fixed by the initial values of the step initial data, while the fact that the DSW fans expand is conveyed by the linear dependence on z of the temporal edges $t_j = \tau_j z$ (see vertical dashed lines in Supplementary Fig. 3). Importantly Eqs. (S4-S6) reduce to Eqs. (7) in the main text, when adopting the same normalization introduced in Fig. 3 of the main paper and in Supplementary Fig. 5, i.e. $r_L^\pm = u_0 \pm 2$, $r_R^\pm = -u_0 \pm 2\sqrt{\rho_0}$. The existence condition reduces, in this case, to $1 + \sqrt{\rho_0} \geq u_0 \geq 1 - \sqrt{\rho_0}$ (cyan area in Fig.3 (d)), for the relevant case $\rho_0 \leq 1$. Possibly, in such domain, the left-going DSW can have a point of cavitation (vacuum point) where the minimum density vanishes, which is found to occur at

$$\tau_0 = \bar{r} - \frac{r_R^+ - r_R^-}{2}\left[1 - \frac{r_L^+ - r_R^+}{r_R^- - 2r_R^+ + r_L^+}\frac{E(m)}{K(m)}\right]^{-1}, \tag{S7}$$

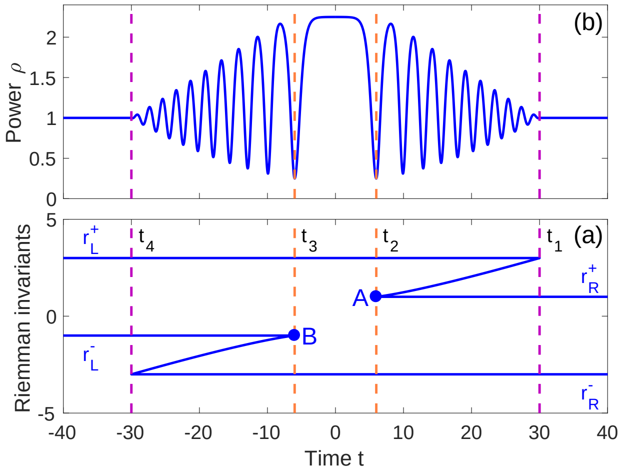

Supplementary Figure 3. DSW-c-DSW, temporal snapshots relative to $z = 12$ of (a) the self-similar structure of Riemann invariants $r^{\pm} = r^{\pm}(\tau)$ of gas dynamic equations (dispersionless NLSE); (b) corresponding construction of normalized power ρ by means of Whitham modulation theory. Vertical dashed lines indicate temporal location of soliton edges ($t_{2,3} = \tau_{2,3}z$, orange lines) and linear edges ($t_{1,4} = \tau_{1,4}z$, magenta lines) of the two DSWs. A,B stand for the vertices which merge in the limit $u_0 \to u_0^{th}$ [Eq. (8) in the main text]. Here $u_0 = 1$, $\rho_0 = 1$.

where $\bar{r} = \frac{r_R^- + r_L^+}{2}$, $K(m)$ and $E(m)$ are elliptic integrals of first and second kind, respectively, of modulus $m = \left(\frac{r_R^+ - r_R^-}{r_L^+ - r_R^+}\right)^2$. The cavitation appears for $m \leq 1$, i.e. above the curve $u_0 = 3\sqrt{\rho_0} - 1$ (red dashed curve in Fig. 3(c) or equivalent Supplementary Fig. 5(d)) corresponding to limit value $m = 1$.

When u_0 increases, the distance between vertices A and B in Supplementary Fig. 3(a) progressively reduces both vertically (since $r_R^+ - r_L^-$ decreases) and horizontally (since the top plateau, with width measured by $\tau_2 - \tau_3$, shrinks). When the threshold value in Eq. (8) in the main text is reached, i.e. in the limit $u_0 \to 1 + \sqrt{\rho_0}$ corresponding to the upper border of cyan region in Fig. 3 in the main, the points A and B in Supplementary Fig. 3(a) collapses ($r_R^+ = r_L^-$ and $\tau_2 = \tau_3 = 0$) and the two DSWs turn out to be directly glued to each other (case not shown). Increasing the chirp u_0 further means that the two DSWs tend to partially overlap around their soliton edges. The only one-phase solution that is compatible with this situation is that the two DSWs become connected through a periodic unmodulated wave, as first pointed out by Bikbaev [11, 12]. In this regime, occurring in general whenever $r_L^+ > r_L^- > r_R^+ > r_R^-$, the

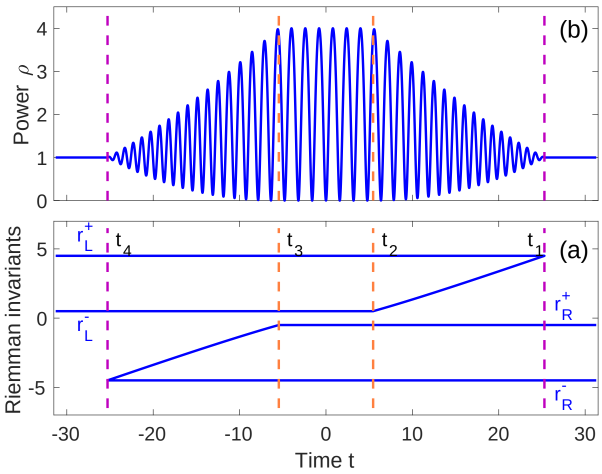

Supplementary Figure 4. DSW-per-DSW: as in Supplementary Fig. 3 when $u_0 > 1 + \sqrt{\rho_0}$ ($r_L^- > r_R^+$), which leads to the folding of Riemann invariants sketched in (a), and the corresponding Whitham construction shown in (b). Here $u_0 = 2.5$, $z = 6$.

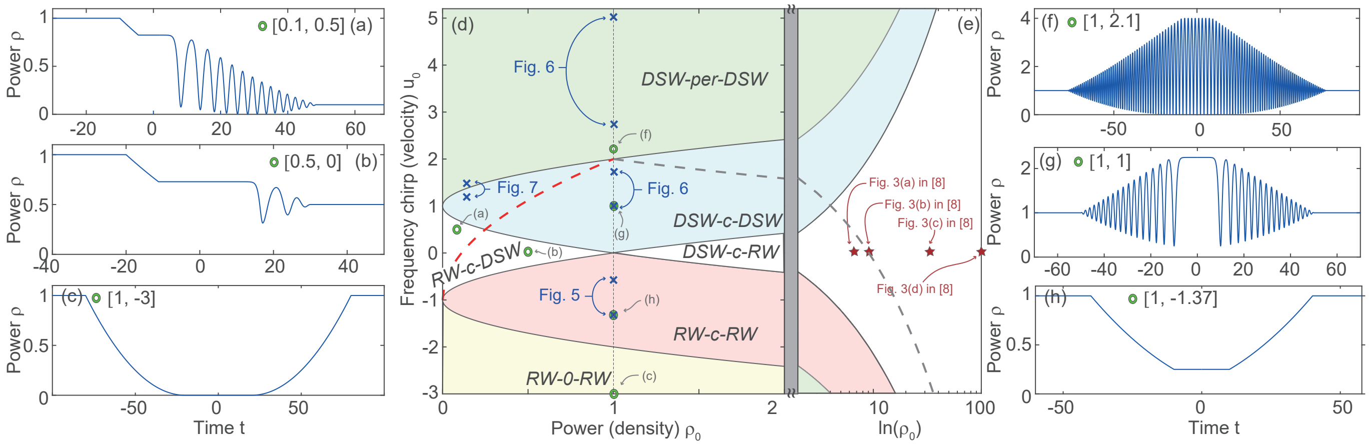

Supplementary Figure 5. Same as Fig. 3 of main text, additionally reporting as red stars the experimental results from Ref. [8] (see also [7]) relative to the region $\rho_0 \geq 2$ reported on a horizontal log scale in (e). (a)-(c) and (f)-(h) are typical examples of output (normalized length $z_L = 20$) from Whitham modulation theory marked with green circles in panel (d). Experimental results are marked with blue crosses. Snapshots [(a) and (b)] illustrate the dam-breaking wave pattern discussed in Ref. [8].

situation illustrated in Supplementary Fig. 3 changes to the one displayed in Supplementary Fig. 4 (see also the video file RiemannNLSmovie.gif for an animation of this and other phase transitions).

In particular, in this case, the constancy of all Riemann invariants in the central region implies that all four Whitham equations have a trivial constant solution, which in turn corresponds to an *unmodulated* periodic wave. In this situation, we find that the external velocities $\tau_{1,4}$ are still given by Eqs. (S4-S6), whereas the location of the points where the DSWs connect to the periodic wave read as

$$\tau_2 = \overline{r} - \frac{r_L^+ - r_L^-}{2}\left[1 - \frac{r_L^+ - r_R^+}{r_L^- - r_R^+}\frac{E(m)}{K(m)}\right]^{-1}, \tag{S8}$$

$$\tau_3 = \overline{r} + \frac{r_R^+ - r_R^-}{2}\left[1 - \frac{r_L^- - r_R^-}{r_L^- - r_R^+}\frac{E(m)}{K(m)}\right]^{-1}, \tag{S9}$$

where $\overline{r} \equiv (r_R^- + r_R^+ + r_L^- + r_L^+)/4$, and $m \equiv \frac{(r_L^+ - r_L^-)(r_R^+ - r_R^-)}{(r_L^+ - r_R^+)(r_L^- - r_R^-)}$. In this regime, in general $m \neq 1$, which means that the DSWs smoothly connect to the periodic nonlinear wave before reaching the local soliton condition ($m = 1$). In other words, in this regime the DSWs no longer include soliton edges, while the unmodulated wave has fully nonlinear character.

Finally, we summarize in Supplementary Fig. 5 all the phase transitions. Supplementary Fig. 5 is similar to Fig. 3 of the main text, except for the fact that, for completness, we also included the experimental results obtained in Ref. [8] (see Supplementary Fig. 5(e), red stars) and relative to the dam breaking or shock tube problem ($u_0 = 0$, step in density). This configuration allowed to investigate the decay into a DSW-RW pair, which is illustrated in panels (a,b) of Supplementary Fig. 5

We also point out, that modulation theory describes equally well the regions in Supplementary Fig. 5 away from the vertical line $\rho_0 = 1$, which represents the piston problem. In this case the left-right symmetry is broken, as shown in the main text. In particular it is worth emphasizing that, for small values of density ratio ($\rho_0 < 0.25$), as used in the experiment, the right DSW is expected to exhibit always cavitation (i.e., a vacuum point) in the whole region of existence of the DSW-c-DSW, and also in the upper region DSW-per-DSW. Indeed such regions lie entirely above the red dashed line that marks the transition to cavitation. This is at variance with the symmetric case $\rho_0 = 1$, where the cavitation marks exactly the transition point ($u_0 = 2$) from the state DSW-c-DSW to the state DSW-per-DSW.

SUPPLEMENTARY NOTE 3: ADDITIONAL NUMERICAL SIMULATIONS

We report few additional numerical simulations to show: (i) why we do not loose generality by considering symmetric steps in the input chirp; (ii) more details on the behavior of the output chirp.

In Supplementary Fig. 6 we compare three different numerical simulations of the spatio-temporal evolution ruled by

the full NLSE, obtained with three input conditions which all lead to the formation of a DSW-c-DSW. In all the simulations we start from a constant input power/density ($\rho = 1$) with a stepwise input in chirp/velocity (pure piston problem) from u_L to u_R. The amplitude of the step is equal in the three cases ($u_L - u_R = 2$), while we vary the average level $u_{av} = (u_L + u_R)/2$ or equivalently the degree of asymmetry of the step. Compared to the symmetric case ($u_L = -u_R = 1$, $u_{av} = 0$) reported in Supplementary Fig. 6(a), Supplementary Fig. 6(b) and (c), relative to $u_{av} = 1$ and $u_{av} = -1$ respectively, show a substantial identical dynamics except for a net tilt (average velocity) of the generated pattern.

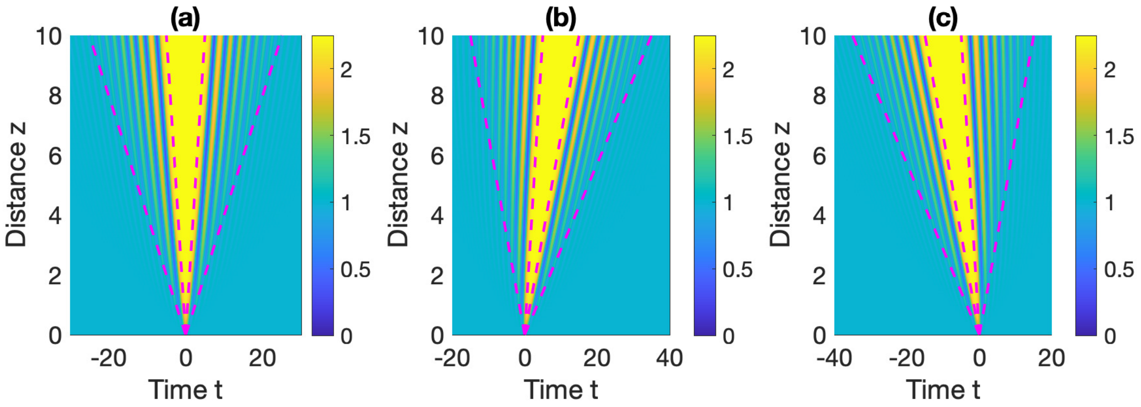

Supplementary Figure 6. Effect of asymmetry in the stepwise input chirp. Comparison of pseudo-color plots of spatio-temporal evolutions obtained from the NLSE (in normalized units) for: (a) $u_L = -u_R = u_0 = 1$ (symmetric chirp, $u_{av} = 0$); (b) $u_L = 2, u_R = 0$ ($u_{av} = 1$); (c) $u_L = -2, u_R = 0$ ($u_{av} = -1$). Here the input density (power) is constant, $\rho = 1$. Dashed lines stand for velocities predicted by Whitham modulation theory [Eqs. (S4-S5-S6)].

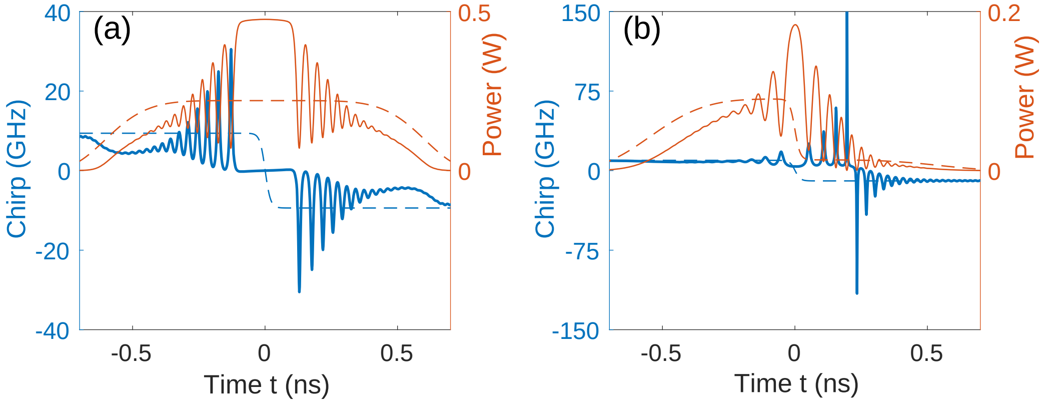

Supplementary Figure 7. Input (dashed curves) and output (solid curves) power and chirp from numerical simulations. (a) Pushing piston case, leading to a DSW-c-DSW solution, corresponding to Fig. 6(a,e) of the main paper. (b) Mixed piston-shock tube problem, leading to an asymmetric DSW-per-DSW solution, corresponding to Fig. 7(d,f) of the main paper.

In Supplementary Fig. 7 we report the input and output power and chirp for two cases involving the generation of DSWs. Supplementary Fig. 7(a) reports in details the case illustrated in Figs. 6(a,e) of the main paper. Overall, the chirp behaves like the power, the only qualitative difference being that it is odd with respect to the time origin. The chirp display local minima (maxima) in correspondence of local maxima (minima) of the power. Supplementary Fig. 7(b) reports the most involved case, corresponding to Figs. 7(d,f) of the paper, where the solution is of DSW-per-DSW type, with a vacuum point on DSW-R ($t > 0$). The main features here are the pronounced spikes around the vacuum point, where the envelope of the chirp diverges. The singularity of the chirp at the vacuum point is not a problem from the physical point of view, because the power goes to zero at the same time. As a general remark, the rapid oscillations associated to a DSW prevent us to perform real time measurements of the chirp.

SUPPLEMENTARY NOTE 4: EXPERIMENTAL SETUP

Pulse shaping with an evolutionary algorithm

The main experimental difficulty encountered during this work was to get the right initial conditions at the input of the HNLF fiber. Indeed, generating the chirp profiles showed in Figures 4 and 7 of the main text require a triangular/M-shaped control pulses with the flattest slope possible. Unfortunately, many experimental factors can degrade these profiles. We can cite among these factors: the distortion of the pulses emitted by the electrical generators (AWGs), the nonlinear distortions introduced by the RF amplifiers, the nonlinear responses of the EOMs and the fiber amplifiers. As an example, the red curve in Supplementary Fig. 8(a) depicts the optical signal generated at the input of the HNLF when a perfect triangular electrical signal is generated by the AWGs. The slopes of the M are not flat which would lead to non-flat frequency chirps along the two sides of the step. Therefore, in order to improve the pulse shapes and insure perfect light profiles in our experiments, we implemented a pre-compensation scheme. This means that we deformed the initial electrical signals (generated upstream by the AWGs) in order to obtain light pulses (emitted downstream by the light amplifiers) having the targeted shapes with the most regular slopes.

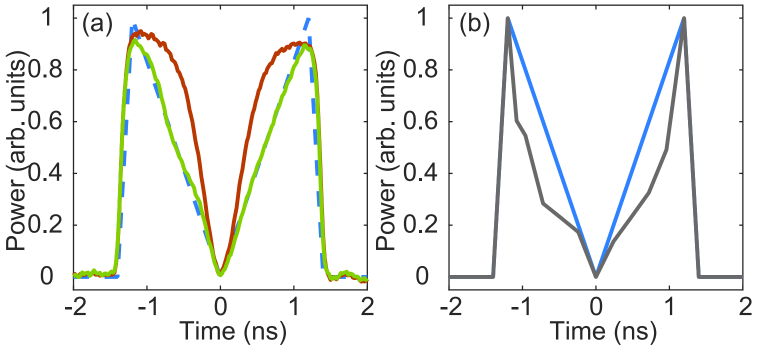

Supplementary Figure 8. (a) Optical signals. The dotted blue line represents the objective curve, the orange curve is without pre-compensation (intensity modulator driven by the blue curve shown in (b)) and the green curve is with pre-compensation (intensity modulator driven by the grey curve shown in (b)). (b) RF signals. The grey curve shows the optimized profile to obtain the optimized optical trace depicted in (a).

Since there is no mean to predict the variations of the introduced nonlinear distortions, we have implemented an inverse resolution algorithm to solve the problem. Our approach is inspired by a previous work on the use of Hill Climbing algorithms [13]. Applied to our particular case, it consists in taking an initial electric profile called father (which of course results in a different light pulse shape from the wanted one) then introducing a random modification on this profile to create a new profile called son. If this modification allows to go closer to the targeted light shape, this modification is kept and the son-profile become the new father. Otherwise, a new modification is applied to the father. The process of modification-evaluation is applied until the son-profile allows for progressively approaching the targeted pulses shapes (M-shaped or triangular). To determine, how much a son-profile is more or less suited than its father, we defined the following fitness function J:

$$J = \sqrt{\sum (\text{Shape} - \text{Shape}_{\text{target}})^2} + F_{\text{factor}} \times \sqrt{\sum (\text{Chirp} - \text{Chirp}_{\text{target}})^2}, \tag{S10}$$

where F_{factor} is the coefficient of pondering of the cost function (curve and derivative) and J represents the degree of fitness. It tends to zero when we approach the exact pulse shape. Note that this function combines both the shape of the pulses and their derivative. Indeed, this allows avoiding local minima and fastening the convergence of the algorithm. After performing several trials, we found that the choice $F_{\text{factor}} = 7$ permits to achieve a satisfactory convergence after a reasonable number of iterations.

An example of the measured vs. the target signal is reported in Supplementary Fig. 8 for the generation of an M-shaped pulse (pushing pistion). The black line in Supplementary Fig. 8(b) shows the electrical profile allowing the formation of M-shaped pulses depicted in green line in Supplementary Fig. 8(a) . This profile has been retrieved thanks to our algorithm after 350 rounds of modification-evaluation. It took a total time of 30 to 40 minutes to execute the algorithm and to achieve an acceptable convergence (decrease in J from 0.3 to 0.055). Note that the modifications made on the AWGs profiles are more noticeable in the center than the edges. Thanks to this algorithm we succeed in generating the perfect initial chirp profiles required in our experiments for both pushing and retracting

pistions. We also noticed during our experiments that the retrieved profiles tend to be stable for a few days before to restart the operation again. This method had also been applied to generate flat intensity profiles at the DCF input.

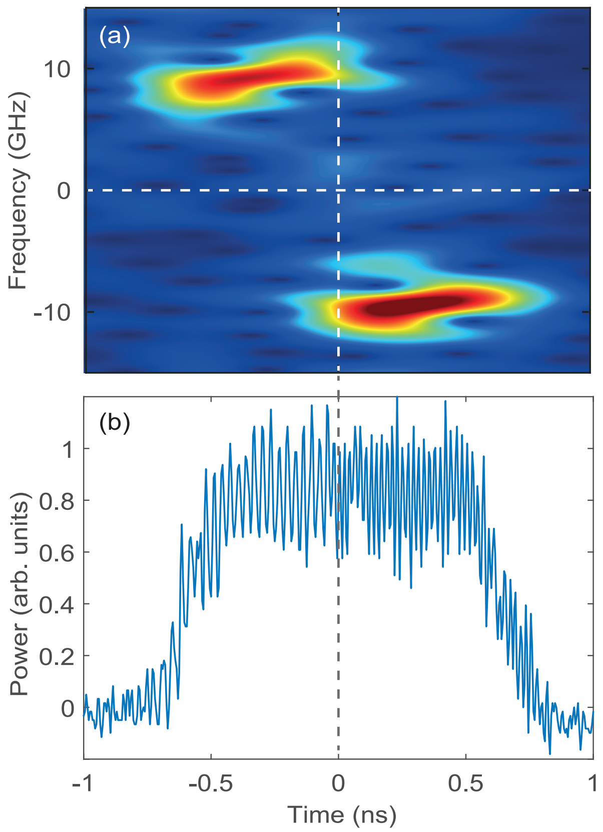

Supplementary Figure 9. Frequency chirp measurements. (a) Spectrogram of Fig. 5 and (b) heterodyne beating trace used to plot it.

Frequency chirp measurements

Supplementary Fig. 9(a) shows a typical experimental spectrogram used to measure frequency chirps of the pulses used in our experiments. It corresponds to the one shown in Fig. 4(b) in the main text. To record these spectrograms, we used a heterodyne detection technique: First, the optical pulses under study are mixed with a local oscillator made of an ultra-narrow laser source of 40 mW delivered by an NKT Basik diode. The bandwidth of this diode is smaller than 100 Hz and its frequency is detuned from the pulses central frequency by about 35 GHz. This frequency is intentionally larger than the chirp excursion in order to avoid spurious interactions between the different frequency lines. Secondly, thanks to a fast-photodiode, we measure the temporal beating between the pulses under study and the local oscillator, as can be shown in Supplementary Fig. 9(b). Finally, we retrieve the spectrogram representation previously showed in Supplementary Fig. 9(a) by computing a sliding window Fourier Transform on this interference figure.

SUPPLEMENTARY REFERENCES

[1] R. J. Leveque, *Finite-Volume methods for Hyperbolic Problems* (Cambrige, 2004).
[2] J. Kevorkian, *Partial Differential Equations, Analytical Solution Techniques*, (Springer, New York, 2000), 2nd ed., p. 364.

[3] M. Conforti, F. Baronio, and S. Trillo, *Resonant radiation shed by dispersive shock waves*, Phys. Rev. A **89**, 013807 (2014).
[4] P. O. K. Krehl, *The classical Rankine-Hugoniot jump conditions, an important cornerstone of modern shock wave physics: ideal assumptions vs. reality*, Eur. Phys. J. **40**, 159 (2015).
[5] G. A. El and M. A. Hoefer, *Dispersive shock waves and modulation theory*, Physica D **333**, 11 (2016).
[6] G.A. El, V.V. Geogjaev, A.V. Gurevich, A.L. Krylov, *Decay of an initial discontinuity in the defocusing NLS hydrodynamics*, Physica D: Nonlinear Phenomena, **87**, 186-192, (1995).
[7] M. Conforti, G. Xu, A. Mussot, A. Kudlinski, and S. Trillo, *Observation of the rupture of a photon dam in an optical fiber*, in *Nonlinear Guided Wave Optics: A testbed for extreme events*, edited by S. Wabnitz, (IOP Publishing, Bristol, 2017), Chap. 4.
[8] G. Xu, M. Conforti, A. Kudlinski, A. Mussot, and S. Trillo, *Dispersive dam-break flow of a photon fluid*, Phys. Rev. Lett. **118**, 254101 (2017).
[9] S. Trillo and M. Conforti, *Shock Waves*, in *Handbook of Optical fibers*, edited by G-D. Peng, (Springer, Singapore, 2019), pp. 411-416.
[10] M. V. Pavlov, *Nonlinear Schrödinger equation and the Bogolyubov-Whitham method of averaging*, Theor. Math. Phys. **71**, 584 (1987).
[11] R. F. Bikbaev, *Large-time asymptotics of the solution of the nonlinear Schrödinger equation with boundary conditions of the step type*, Teor. Mat. Fiz. **81**, 3 (1989);
[12] R. F. Bikbaev, *Finite-gap attractors and transition processes of the shock-wave type in integrable systems*, J. Math. Sci. **77**, 3033 (1995).
[13] F. Braud, A. Bendahmane, A. Mussot, and A. Kudlinski, *Simultaneous control of the wavelength and duration of Raman-shifting solitons using topographic photonic crystal fibers*, J. Opt. Soc. Am. B **32**, 2146-2152 (2015).
