# Supplementary material for: The piston Riemann problem in a photon superfluid
Source: Nat Commun. 2022 Jun 6;13:3137. doi: 10.1038/s41467-022-30734-5 (PMC9170689; doi:10.1038/s41467-022-30734-5)
Supplement: Supplementary file 3 — Description of Additional Supplementary Files [file 41467_2022_30734_MOESM3_ESM.pdf]

File name: Supplementary Movie 1

Description: The gif file RiemannNLSmovie.gif reports the animation of the phase transitions of the photonic flow, as obtained from Whitham modulation theory. In particular the animation reports, similarly to Supplementary Figs. 3 and 4, both the Riemann selfsimilar construction and the corresponding temporal pattern of output power (density) $\rho(t,\overline{z})$ at fixed normalized distance $\overline{z}=8$, as the amplitude of the initial step in frequency chirp u_0 changes from negative to positive values across different phase transitions. Here the step initial condition is that of the pure piston problem with step boundaries $\rho_L=\rho_R \equiv \rho_0=1$, $u_L=-u_R=u_0$, and the movie also shows the corresponding location of the input on the parameter plane (ρ_0, u_0) along the vertical cut $\rho_0=1$ as in Fig. 3 of the main paper and in Supplementary Fig. 5. The results of such construction has been verified to be in good quantitative agreement with numerical integration of the NLSE.

Interestingly enough, above threshold for the onset of the DSW-per-DSW, increasing u_0 results only in a wider temporal extension of the portion occupied by the unmodulated periodic wave, while its extremes remain locked to $\rho_{min}=0, \rho_{max}=4$. Finally, we point out that the patterns in the movie are not directly comparable with the experiment, since in the latter the output is at constant physical length which corresponds to a normalized length $\overline{z}$ which varies with power (or equivalently with dimensionless input step amplitude u_0).
