# Supplementary figures and images for: The piston Riemann problem in a photon superfluid
Source: Nat Commun. 2022 Jun 6;13:3137. doi: 10.1038/s41467-022-30734-5 (PMC9170689; doi:10.1038/s41467-022-30734-5)

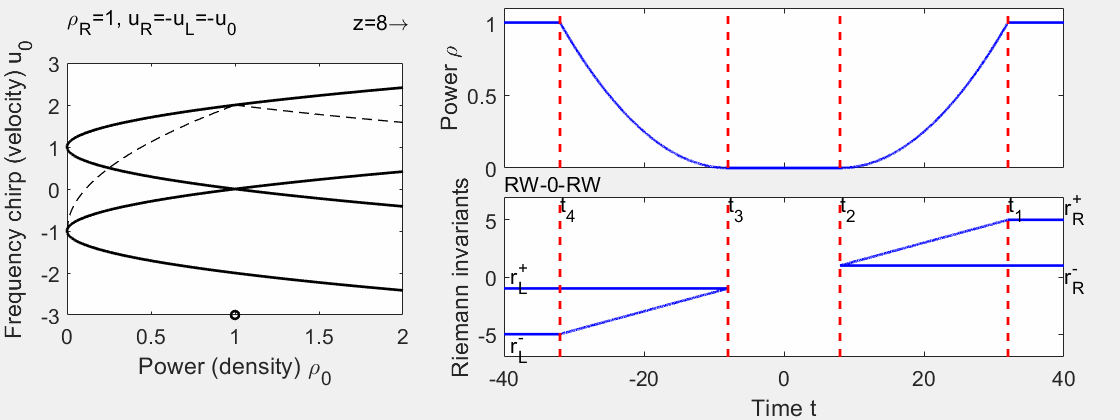

Supplement: Supplementary file 4 — Supplementary Movie 1 [file 41467_2022_30734_MOESM4_ESM.gif]
